# Spousal and living related kidney transplantation: our center experience

**DOI:** 10.1186/s12893-021-01447-1

**Published:** 2021-12-29

**Authors:** Utku Ozgen, Murat Ozban, Onur Birsen, Sevda Yilmaz, Belda Dursun, Mevlut Ceri, Mesut Eker, Huseyin Cagatay Aydin

**Affiliations:** 1 Department of Surgery, School of Medicine, Pamukkale University, Denizli, Turkey; 2 Department of Nephrology, School of Medicine, Pamukkale University, Denizli, Turkey; 3 Department of Surgery, School of Medicine, Medipol University, Istanbul, Turkey

**Keywords:** Spousal, Living-related, Kidney transplantation

## Abstract

**Background:**

Kidney transplantation is the most preferred type of renal displacement therapy for end stage renal disease (ESRD) patients. More patients developed ESRD. The most important source is the donations from unrelated spouses. In this study, we aimed to compare the transplantation data obtained from the spouses of the patients with the transplantation data obtained from other relatives.

**Methods:**

The data including 167 living kidney transplantations performed between January 2006 and December 2019 were retrospectively collected. The patients were divided into two groups; spousal donor group (n: 53) and living-related donor group (n: 114).

**Results:**

There was no significant difference in delayed graft function in both groups. There were no patients with acute rejection proven by biopsy or considered biochemically in the spousal donor group. With regard to 3-year results in the living-related donor group the patient survival rate was 100%, while it was 98.2% in terms of graft survival.

**Conclusions:**

In conclusion, similar patient and graft survival rates between spousal donor kidney transplantation and living-related kidney transplantation has made spousal donor kidney transplantation, with possible problems in terms of tissue compatibility, an acceptable alternative to donor supply.

## Introduction

The number of patients diagnosed with end-stage renal disease (ESRD) is increasing, in which kidney transplantation is the most common type of renal replacement therapy. As more patients develop end-stage renal disease (ESRD) [1], the issues faced in identifying donor organs lead to problems, increasing the number of cadaveric transplants. In many countries, the donor organs come primarily from living donors, among which living-related donors (LRDs) remain the main source. In our country, donations by relatives of up to the fourth degree are allowed without the need for ethics committee approval, while the most important sources are unrelated spouses. In the present study, we compare the data related to transplants from spouses with the data from other relatives.

## Materials and methods

Data related to 169 living kidney transplants performed between January 2006 and December 2019 were collected retrospectively, and the cases were divided into spousal donor transplant (n = 53) and living-related donor transplant (n = 116) groups. The garnered demographic data included age, sex, HLA mismatch, length of preoperative dialysis and body mass index (BMI), while the medical data included post-transplant graft and patient survival, serum creatinine levels, delayed graft function and the presence of biopsy-proven acute rejection. The applied immunosuppressive therapy protocol included the preoperative initiation of corticosteroids and mycophenolate mofetil, and intraoperative basiliximab induction, and the recipients were administered 500 mg of intravenous methylprednisolone before reperfusion. In the cases in which transplants were made from living donors, an additional standard dose of tacrolimus therapy was administered to the recipient in cases of creatinine levels of < 3. The same standard therapy was continued as a maintenance treatment protocol for the patient. The target for tacrolimus was to maintain an FK 506 trough level of 8–10 ng/ml. Prednisolone was initiated at a dose of 100 mg on the postoperative 1st day, and was reduced by 10 mg every day until a dose of 20 mg/day was reached.

A written informed consent was obtained from the patients and from healthy participants. The study protocol was approved by the Pamukkale University Ethics Committee. The study was conducted in accordance with the principles of the Declaration of Helsinki.

### Statistical analysis

SPSS 22.0 (IBM Corp., Armonk, NY, USA) was used for performing istatistical analysis. Analytical characteristics were given as percentage, mean and SD, or median. The chi-square test was used for univariate analysis of categorical variables. Values of p < 0.05 were considered to be statistically significant.

## Results

Table 1 presents the demographic data, as well as data on the immunosuppressive therapy, length of follow-up, rate of HLA mismatch, rates of function and rejection, body mass indices, graft and patient survival rates of the 53 patients in the spousal donor group and the 116 patients in the living-related donor group. The mean age of the patients was 49.73 years in the spousal donor transplant group and 31.45 years in the living-related donor transplant group. All of the transplants were the first transplant surgery undergone by the patients aside from one case. Only one of our patients underwent a third transplantation, having previously received a kidney from both parents (mother and father, respectively), with approximately 12 years of function for both grafts. The patient’s spouse was the donor for the third transplant. The patient with the 5/6 mismatch developed no immunological complication during or after the transplantation, and their creatinine value was 0.96 mg/dl in the postoperative 2nd year. They remain under follow-up. The HLA mismatching rate was 5.06 for the recipients in the spousal donor transplant group and 3.086 for those in the living-related donor transplant group. There were 19 (35.8%) full-mismatch patients. No significant difference was identified in the delayed graft functions of the two groups. There were no patients with biopsy-proven or biochemically-suspected acute rejection in the spousal donor transplant group. Biopsy-proven acute rejection was detected in only one patient in the living-related donor transplant group. This patient, who underwent treatment, recorded a creatinine level of 1.8 mg/dl which has been maintained at healthy levels 2 years after follow-up. The length of follow-up of the patients in the present study ranged from 6 months to 14 years. In our 14-year experience, the cumulative rates of patient and graft survival were 96.3% and 96.3% in the spousal donor transplant group, and 97.5% and 91.6% in the living-related donor transplant group, respectively. Our 3-year rates, as more cross-sectional data, in turn, revealed patient and graft survival rates of 100% and 98.1%, respectively in the spousal donor transplant group. In this group, the graft loss resulted from renal artery thrombosis, which was the first case identified in the series. In the living-related donor transplant group, our 3-year results were 100% for patient survival and 98.2% for graft survival. An analysis of the serum creatinine levels of the recipients with functional grafts at various times after transplantation is presented in Table 2, in which no significant difference in serum creatinine levels can be identified between the two groups. No minor or major complications were identified during the follow-up of all donors in either group.

**Table 1:** Clinical characteristics of transplants from spousal donors and living-related donors

	SD group (n:53) median	LR group (n:116) median	P value
Recipient age (years)	49.73	31.45	< .001
Donor age (years)	44.36	56.14	< .001
Follow-up period (month)	59.18	71.12	ns
Recipients’ BMI (kg/m^2^)	27.12	28.96	< .001
HLA mismatches	5.06	3.08	< .001
Pre-tx dialysis period (month)	24.17	10.91	< .001
Acute rejection	0	1(0.9%)	ns
DGF	3(5.6%)	5(4.3%)	ns
Patient death (cumulative)	2(3.7%)	3(2.5%)	ns
Graft loss (cumulative)	2(3.7%)	11(9.4%)	< .001
Patient death (first 3 years)	0	0	ns
Graft loss (first 3 years)	1(1.8%)	2(1.8%)	ns

*SD* spousal donor, *LR* living related

**Table 2:** Living-related donor group data

	Donor	Kinship status	Relationship degree
1.	FK	Mother	1
2.	ET	Mother-in-law	1
3.	GO	Mother	1
4.	ME	Mother	1
5.	RÇ	Father	1
6.	AG	Mother	1
7.	HE	Brother	2
8.	EB	Sister	2
9.	FA	Mother-in-law	1
10.	AG	Father	1
11.	TT	Cousin	4
12.	GK	Sister	2
13.	FA	Mother	1
14.	EA	Father	1
15.	MT	Brother	2
16.	İK	Brother	2
17.	AA	Father	1
18.	AT	Mother	1
19.	NA	Mother	1
20.	EK	Mother	1
21.	Aİ	Father	1
22.	AT	Father	1
23.	İS	Father	1
24.	MK	Father	1
25.	İF	Uncle	3
26.	ÜO	Mother	1
27.	MA	Father	1
28.	TT	Brother	2
29.	SA	Mother	1
30.	SG	Mother	1
31.	FD	Aunt	3
32.	FT	Mother	1
33.	MK	Uncle	3
34.	AA	Father	1
35.	GC	Mother-in-law	1
36.	HE	Sister-in-law	2
37.	CT	Father	1
38.	MG	Father	1
39.	NZ	Mother	1
40.	AB	Mother	1
41.	DA	Father	1
42.	SK	Mother	1
43.	HZ	Mother	1
44.	İS	Brother	2
45.	HG	Mother	1
46.	AB	Sister	2
47.	İY	Father	1
48.	YS	Uncle	3
49.	AH	Mother	1
50.	SA	Father	1
51.	EK	Mother	1
52.	HP	Father	1
53.	SÇ	Mother	1
54.	HG	Mother	1
55.	ÜA	Sister	2
56.	ET	Mother	1
57.	FY	Mother	1
58.	HG	Mother	1
59.	PK	Mother	1
60.	ÖI	Sister	2
61.	HC	Mother	1
62.	İG	Father	1
63.	YT	Father	1
64.	OÇ	Son	1
65.	İŞ	Father	1
66.	MH	Mother	1
67.	SÖ	Mother	1
68.	MV	Father	1
69.	ÖC	Brother	2
70.	EC	Son	1
71.	HÇ	Mother	1
72.	MA	Cousin	4
73.	HÖ	Sister	2
74.	YH	Mother	1
75.	İÇ	Father	1
76.	Mİ	Father	1
77.	AÇ	Father	1
78.	BB	Father	1
79.	RA	Grandmother	2
80.	ÜÖ	Mother	1
81.	EK	Sister	2
82.	NT	Father	1
83.	AD	Brother	2
84.	ME	Father	1
85.	AŞ	Brother	2
86.	MK	Mother	1
87.	MK	Son	1
88.	ME	Mother	1
89.	DE	Mother	1
90.	AY	Brother	2
91.	SÜ	Father	1
92.	YB	Father	1
93.	YY	Father	1
94.	EY	Father	1
95.	AY	Brother	2
96.	FD	Mother	1
97.	FK	Sister	2
98.	ÜD	Sister	2
99.	MÇ	Father	1
100.	SA	Mother	1
101.	ZG	Sister	2
102.	SA	Sister	2
103.	EÖ	Mother	1
104.	MY	Brother	2
105.	OÖ	Brother	2
106.	ST	Sister	2
107.	JŞ	Mother	1
108.	HÇ	Father	1
109.	NE	Mother	1
110.	SA	Mother	1
111.	ŞY	Brother	2
112.	SD	Mother	1
113.	KA	Sister	2
114.	ZE	Mother	1

## Discussion

The advanced immunosuppressive therapy approaches adopted over the last two decades have led to a rapid increase in the success rates of kidney transplantation [2]. Unfortunately, the number of cadaveric transplantations has not accelerated to any significant degree, and the number of transplant candidates is rapidly rising, leading to an increasing need for organs [3] and prolonged waiting times for transplants [4]. Several transplant centers are experiencing both tissue and blood group compatibility issues, especially those performing frequent cross-over transplantations, leading to a greater preference for spousal donor transplants in many centers [5, 6]. This group, in which both tissue and blood group compatibility issues are common, has become the preferred alternative, particularly due to the high rate of consanguineous marriages in our country. According to the reports of many centers worldwide, graft survival rates are equal to that of transplants from single haplotype-matched living donors, and the graft and patient survival rates are better than with cadaveric transplants [7].

In the present study, children who received kidneys from their parents accounted for the majority of transplant patients in the living-related donor transplant group, explaining the lower mean age of the recipients and the higher mean age of donors in this group. Young age is considered a risk factor for a higher incidence of rejection in young people with a stronger immunological structure than in others [8]. Addressing this issue, Gjertson et al. [9] compared spousal and other genetically unrelated donor transplants, and concluded that graft survival rates were almost the same in both groups. Recent reports in literature suggest that the outcome of transplants is not affected much by HLA group mismatches [10], while several single-center studies have reported graft survival rates to be similar in both living-related and spousal donor groups, but with more HLA mismatches in the spousal donor group [11]. The present study, despite the significantly higher HLA mismatch rate in the spousal donor transplant group than in the living-related donor transplant group, identified no adverse effect of HLA mismatch on outcomes. Both groups had stable postoperative serum creatinine levels, which were usually higher in the spousal donor transplant group at each post-transplant time point than in the living-related donor transplant group, although the difference was statistically insignificant.

The findings of the present study suggest that the 3-year survival rates of both the patient and graft were quite high in both groups. The most important factor contributing to this result is the stringent decision-making mechanism applied by our center in regards to transplants with marginal criteria, as well as the stable immunosuppressive therapy protocol with basiliximab induction applied, which is uncommon in many centers. Similar patient and graft outcomes among spousal and related allografts have been reported also in Caucasian [12] and Japanese [13] patients, which it is believed can be attributed to strong immunosuppression, high-quality living grafts, spouses of similar age and better drug regimen adherence as a result of the recipient and donor living together [14].

## Conclusion

In conclusion, the similar patient and graft survival rates in the spousal and living-related donor kidney transplant groups suggest that spousal donor kidney transplants, in which tissue compatibility issues may arise, are an acceptable alternative for donor supply.
